# Polygenic approaches to detect gene–environment interactions when external information is unavailable

**DOI:** 10.1093/bib/bby086

**Published:** 2018-09-13

**Authors:** Wan-Yu Lin, Ching-Chieh Huang, Yu-Li Liu, Shih-Jen Tsai, Po-Hsiu Kuo

**Affiliations:** 1 Institute of Epidemiology and Preventive Medicine, College of Public Health, National Taiwan University, Taipei, Taiwan; 2 Department of Public Health, College of Public Health, National Taiwan University, Taipei, Taiwan; 3 Center for Neuropsychiatric Research, National Health Research Institutes, Miaoli County, Taiwan; 4 Department of Psychiatry, TaipeiVeterans General Hospital, Taipei, Taiwan; 5 Division of Psychiatry, National Yang-Ming University, Taipei, Taiwan

**Keywords:** diastolic blood pressure, systolic blood pressure, gene–alcohol interaction, gene–smoking interaction, Taiwan Biobank, multiple-testing correction

## Abstract

The exploration of ‘gene–environment interactions’ (G × E) is important for disease prediction and prevention. The scientific community usually uses external information to construct a genetic risk score (GRS), and then tests the interaction between this GRS and an environmental factor (E). However, external genome-wide association studies (GWAS) are not always available, especially for non-Caucasian ethnicity. Although GRS is an analysis tool to detect G × E in GWAS, its performance remains unclear when there is no external information. Our ‘adaptive combination of Bayes factors method’ (ADABF) can aggregate G × E signals and test the significance of G × E by a polygenic test. We here explore a powerful polygenic approach for G × E when external information is unavailable, by comparing our ADABF with the GRS based on marginal effects of SNPs (GRS-M) and GRS based on SNP × E interactions (GRS-I). ADABF is the most powerful method in the absence of SNP main effects, whereas GRS-M is generally the best test when single-nucleotide polymorphisms main effects exist. GRS-I is the least powerful test due to its data-splitting strategy. Furthermore, we apply these methods to Taiwan Biobank data. ADABF and GRS-M identified gene × alcohol and gene × smoking interactions on blood pressure (BP). BP-increasing alleles elevate more BP in drinkers (smokers) than in nondrinkers (nonsmokers). This work provides guidance to choose a polygenic approach to detect G × E when external information is unavailable.

## Introduction

Evidence of ‘gene–environment interactions’ (G × E) has been found in some phenotypes and complex diseases [1–5]. Genes and environmental exposure may jointly influence disease liability. The identification of G × E is important to improve the accuracy and precision of assessing genetic and environmental influences [6]. The G × E identification is an active research area that generates high expectations, but usually leads to great disappointment [7]. With the shift toward genome-wide association studies (GWAS), genome-wide G × E studies are fairly common in human genetic research [8]. However, few significant and replicated G × E have been found from GWAS to date [9–12]. Popular choices for genome-wide G × E analyses include single marker analysis and set-based (or gene-based) analysis [13–16]. However, with the large number of single-nucleotide polymorphisms (SNPs) or genes in the human genome, both approaches suffer from low statistical power due to a harsh penalty from multiple hypothesis correction.

Many G × E studies avoid the harsh multiple-testing penalty by constructing a genetic risk score (GRS) according to previous GWAS findings and then testing the significance of the interaction term (GRS × E) in a regression model [3–5, 17–19]. The GRS or polygenic risk score (PRS) is commonly used to summarize genetic effects among an ensemble of SNPs that do not individually achieve the genome-wide significance level, i.e. $5\kern0.5em \times \kern0.5em {10}^{-8}$ [20–22]. Information on *L* disease-associated SNPs that are nearly in linkage equilibrium is aggregated by defining a GRS of the *i*^th^ subject as ${GRS}_i=\kern0.5em {\sum}_{l=1}^L{\widehat{\beta}}_l{g}_{il}$, where ${g}_{il}$ is the number of risk alleles (those associated with disease liability according to previous studies) at the *l*^th^ SNP of that subject and ${\widehat{\beta}}_l$ is the weight given to the *l*^th^ SNP ($l = 1,\cdots, L;\, i=1,\cdots, n$, where *n* is the sample size).

If all the ${\widehat{\beta}}_l$s are specified to be 1, the ${GRS}_i$ is the overall genetic burden of the *i*^th^ subject by summing his/her total number of risk alleles. For example, the gene × physical activity interaction in obesity was identified using an unweighted GRS [3, 4]. This unweighted GRS was composed of 12 body mass index (BMI) associated SNPs that were discovered by previous GWAS [23–27]. However, the unweighted GRS (all ${\widehat{\beta}}_l$s = 1) should be used only if the *L* disease-associated SNPs are considered of equal importance.

When a weighted GRS is considered, researchers often extract ${\widehat{\beta}}_l$s from external GWAS that focus on the same ethnicity [28]. Because GWAS usually fit a regression model for each SNP and provide summary statistics for the scientific community, ${\widehat{\beta}}_l$s come from the regression estimates regarding SNP effects. For a continuous trait, ${\widehat{\beta}}_l$ ($l = 1,\cdots, L$) is the effect size of the risk allele at the *l*^th^ SNP [5]. For a binary trait, ${\widehat{\beta}}_l$ is the log odds ratio (OR) of the risk allele at the *l*^th^ SNP [19, 29–31]. For example, the interactions of gene × physical activity, gene × alcohol consumption and gene × socioeconomic status were detected using a weighted GRS [5] composed of 94 BMI-associated SNPs that were identified by a previous GWAS [32]. Mullins *et al.* found that childhood trauma has a greater effect in subjects with lower genetic liability for major depressive disorder [19], by constructing a weighted GRS using results from the Psychiatric Genomics Consortium [33, 34]. These G × E findings rely on previous GWAS discoveries. However, an appropriate external GWAS is not always available, especially for non-Caucasian ethnicity.

When there is no appropriate external information, the weights have to come from the current study. Hüls *et al*. have proposed a ‘GRS-marginal-internal approach’ and a ‘GRS-interaction-training approach’ for pathway-oriented G × E studies [28, 35]. That is, G × E interactions are investigated in a pre-selected set of candidate SNPs, e.g. SNPs from a biological pathway. The weights of these SNPs are then estimated by a multivariate elastic net regression [36]. However, take Taiwan Biobank (TWB) GWAS as an example, there are 601531 autosomal SNPs passing the quality control stage. After pruning SNPs in high linkage disequilibrium (LD), we still have 143574 SNPs. Fitting a multivariate elastic net regression on such a large number of SNPs is computationally infeasible. Therefore, in this work, we borrow the idea from Hüls *et al*. [28, 35] and list two GRS-based tests that can be implemented in GWAS.

The GRS weights can be determined in the following two ways: (1) based on SNP marginal effects (we call it ‘GRS-M’) and (2) based on SNP × E interaction effects (we call it ‘GRS-I’). The abovementioned [3–5, 19] and many other G × E studies [17, 18, 29–31, 37–39] were all applications of the GRS-M approach, because their GRSs were constructed by SNPs with larger marginal effects on the phenotype. In most GRS-M applications, the weights ${\widehat{\beta}}_l$s ($l=1,\cdots, L$) required to build GRS came from external studies [3–5, 17–19, 29–31].

Another way to construct a GRS for detecting G × E is to use the weights from SNP × E interaction effects themselves, and we call it the GRS-I approach. To preserve the validity, GRS-I should be performed by splitting the whole sample into a training subset and a testing subset. The weights ${\widehat{\beta}}_l$s are decided by the regression coefficients of SNP × E interaction term using the training subset. Then, GRS-I is calculated for the testing subset, and the significance of GRS-I × E is assessed.

Although G × E have been found for several phenotypes, many interaction effects may remain hidden due to the lack of a powerful polygenic test. In this work, we use the ‘adaptive combination of Bayes factors method’ (ADABF) [40] to aggregate G × E signals and to test the significance of G × E by a polygenic test. More than GRS-M and GRS-I, if the ADABF test result is significant, we can further pinpoint individual SNPs that interact with E. We compare the performance of ADABF, GRS-M and GRS-I with extensive simulations. Regarding the ability to pinpoint individual SNP × E, we calculate the positive predictive value (PPV) and sensitivity of ADABF and compare these values with those of single marker analysis. We then apply these approaches to TWB data to explore gene × alcohol consumption and gene × smoking interactions on blood pressure (BP) levels.

## Methods

### Adaptive combination of Bayes factors method

#### A pruning stage:

There are a pruning stage and a screening stage prior to using ADABF. Both the two stages are also commonly used in GRS methods [12, 20, 22, 41]. We first prune SNPs in high LD to eliminate a large degree of redundancy in SNPs. Suppose we have a GWAS dataset called ‘TWBGWAS’, the PLINK command ‘plink --bfile TWBGWAS --chr 1-22 --indep 50 5 2’ is used to prune SNPs in high LD [42]. This command removes SNPs with a variance inflation factor (VIF) > 2 within a sliding window of size 50. The sliding window is shifted at each step of five SNPs. VIF is calculated by ${\left(1-{R}^2\right)}^{-1}$, where ${R}^2$ is the multiple correlation coefficient when an SNP is regressed on all other SNPs simultaneously. A VIF of 1 indicates that ${R}^2$ = 0 and the SNP is completely independent of all other SNPs. According to the PLINK guideline of SNP pruning (http://zzz.bwh.harvard.edu/plink/summary.shtml#prune), a VIF between 1.5 and 2 should be used in practice.

#### A screening stage:

Moreover, to improve the statistical power of G × E tests, the remained SNPs are then screened according to their marginal associations with the phenotype. The generalized linear model (GLM) for the *l*^th^ SNP ($l=1,\cdots, L$) is described as follows:

(1) \begin{equation*} g\left[E\left({Y}_i\right)\right]={\beta}_0+{\beta}_{G_l}{G}_{il}+{\boldsymbol{\beta}}_X^{\prime }{X}_i,i=1,\cdots, n, \end{equation*}

where $g\left[\cdot \right]$ is the link function; ${Y}_i$ is the phenotype, ${G}_{il}$ is the number of minor alleles at the *l*^th^ SNP (0, 1 or 2) and ${X_{i}}$ is the vector of covariates of the *i*^th^ subject. In this screening stage, we test ${H}_0 :{\beta}_{G_l} = 0$ versus ${H}_1:{\beta}_{G_l}\ne 0$ ($l = 1, \cdots, L$). The SNPs passing the screening at the desired significance level (*P* < 0.05) are then analyzed using ADABF. This screening stage that reduces the number of SNPs tested for interactions can substantially increase the power of genome-wide G × E studies [8, 12, 43–48].

Suppose that in a GWAS there are *L* autosomal SNPs retained after the pruning and screening stages. We assess the interaction between the *l*^th^ SNP ($l = 1,\cdots, L$) and E by the following GLM:

(2) \begin{equation*} g\left[E\left({Y}_i\right)\right]={\beta}_0+{\beta}_{G_l}{G}_{il}+{\beta}_E{E}_i+{\beta}_{GE_l}{G}_{il}{E}_i+{\boldsymbol{\beta}}_X^{\prime }{\boldsymbol{X}}_i,i=1,\cdots, n; \end{equation*}

where ${E}_i$ is the environmental factor (E) of the *i*^th^ subject, and the other notations have been described under Equation (1). Let ${\widehat{\beta}}_{GE_l}$ be the maximum likelihood estimate (MLE) of ${\beta}_{GE_l}$. According to the asymptotic normality of MLE, ${\widehat{\beta}}_{GE_l}$ follows a normal distribution with a mean of ${\beta}_{GE_l}$ and a variance of ${V}_l$, i.e. ${\widehat{\beta}}_{GE_l}\sim N\left({\beta}_{GE_l},{V}_l\right)$.

Sizes of interaction effects (${\beta}_{GE_l}$s) depend heavily on the scale of an E. An ${E}_i$ ranging from 0 to 1 and an ${E}_i$ ranging from 0 to 100 should be linked to different prior distributions of ${\beta}_{GE_l}$s. To propose a prior that can be used in most situations, we first rescale ${E}_i$ to range from 0 to 1. Therefore, ${G}_{il}{E}_i$ will be between 0 and 2, the same as ${G}_{il}$. In Equation (2), a binary ${E}_i$ (e.g. smoking versus nonsmoking) is coded as 1 or 0 and a continuous ${E}_i$ is first scaled to a range from 0 to 1. Let ${E}_{\mathrm{min}}$ and ${E}_{\mathrm{max}}$ be the minimum and maximum of a continuous ${E}_i$, where $i= 1, \cdots, n$. The continuous ${E}_i$ is scaled to be ${E_i}^{\prime} = \left({E}_i - {E}_{\mathrm{min}}\right)\!/\!\left({E}_{\mathrm{max}} - {E}_{\mathrm{min}}\right)$, where $i = 1,\cdots, \kern0.5em n$.

The Wellcome Trust Case Control Consortium (WTCCC) GWAS specified a normal distribution with a mean of 0 and a variance of *W* = 0.2^2^ = 0.04 as the prior of SNP main effects, i.e. ${\beta}_{G_l}\sim N\left(0,\kern0.5em W\kern0.5em =\kern0.5em 0.04\right)$. Because ${G}_{il}{E}_i$ has been scaled to range from 0 to 2 (the same as ${G}_{il}$), we may consider the appropriateness of using $N\left(0,W=0.04\right)$ as the prior of ${\beta}_{GE_l}$s. Similarly with SNP main effects, most reported SNP × E interactions are of modest effect sizes that can be positive or negative [49–51]. $N\left(0,\kern0.5em W\kern0.5em =\kern0.5em 0.04\right)$ may be a reasonable prior for ${\beta}_{GE_l}$s as well. In a binary trait analysis such as the WTCCC GWAS [52], this prior implies that we believe 95% of ORs range from $\exp \kern0.5em \left(-2 \times 0.2\right) = 0.67$ to $\exp \kern0.5em \left(2\kern0.5em \times \kern0.5em .2\right) = 1.49$. For continuous traits, this prior implies that 95% of ${\beta}_{GE_l}$s range from −.4 to 0.4. We consider this prior suitable for a standardized continuous trait with a mean of 0 and an SD of 1. Therefore, for a continuous trait analysis, our R code (http://homepage.ntu.edu.tw/∼linwy/ADABFGEPoly.html) standardizes the trait before implementing the ADABF method.

The prior variance, *W* = 0.2^2^ = 0.04 is originally designed for SNP main effects. Empirical evidence has shown that SNP × E interaction effects are usually modest [49–51], and therefore this prior variance may be slightly large for ${\beta}_{GE_l}$s. However, a larger prior variance can reflect our uncertainty of the prior information [53]. If investigators believe that very few SNP × E interactions may exist in their own study, they can specify a smaller prior variance that provides more shrinkage toward zero and favors more coefficients to be zero [53].

To test whether the *l*^th^ SNP interacts with E, the hypothesis is ${H}_{0,l}:{\beta}_{GE_l}=0$ versus ${H}_{1,l}:{\beta}_{GE_l}\ne 0$ ($l=1,\cdots, L$). The BF is described as follows [54, 55]:

(3) \begin{equation*} {BF}_l=\frac{\Pr \left( Data|{H}_{1,l}\right)}{\Pr \left( Data|{H}_{0,l}\right)}=\sqrt{\frac{{\widehat{V}}_l}{{\widehat{V}}_l+W}}\exp \left(\frac{{\widehat{\beta}}_{GE_l}^2W}{2{\widehat{V}}_l\left({\widehat{V}}_l+W\right)}\right),l=1,\cdots, L, \end{equation*}

where ${\widehat{\beta}}_{GE_l}$ and ${\widehat{V}}_l$ have been estimated from the GLM in Equation (2).

The hypothesis of interest in ADABF is ${H}_0:{\beta}_{GE_1}\!=\!\cdots \!={\beta}_{GE_L}\!\!=0$ (none of the *L* SNPs interact with E) versus ${H}_1:$ at least one ${\beta}_{GE_l}\ne 0$ for $l = 1,\cdots, \kern0.5em L$. ADABF tests the interaction between E and all the *L* SNPs, by combining Bayes factors (BFs) of the *L* SNP × E signals. After obtaining ${BF}_1,\cdots, \kern0.5em {BF}_L$ from Equation (3), we sort these *L* BFs from largest to smallest and denote them as ${BF}_{(1)}\ge {BF}_{(2)}\ge \cdots \ge {BF}_{(L)}$. We calculate a summary score that aggregates the leading *k* BFs, ${S}_k={\sum}_{l=1}^k\log \left({BF}_{(l)}\right)$, where $k=1,\cdots, L$. We then perform *B* resampling replicates, e.g. *B* = 1000 in our simulation and *B* = 10^5^ in the following real data analysis. In each resampling, we draw an *L*-length vector of ${\widehat{\boldsymbol{\beta}}}_{GE,{H}_0}$ containing the MLEs of ${\beta}_{GE_l}$s ($l=1,\cdots, L$) under ${H}_0:{\beta}_{GE_1}=\!\cdots\!={\beta}_{GE_L}=0$. With the typical large sample size of a GWAS, the asymptotic normality of MLE holds so that ${\widehat{\beta}}_{GE_l}$ follows a normal distribution with a mean of 0 (under *H_0_*). Therefore, ${\widehat{\boldsymbol{\beta}}}_{GE,{H}_0}$ follows the multivariate normal distribution ${\boldsymbol{N}\,(\boldsymbol{0}_{L\times 1},\boldsymbol{V}_{L\times L})}$. The (*i*, *j*)^th^ element of the variance–covariance matrix ${\boldsymbol{V}}_{L\kern0.5em \times \kern0.5em L}$ is ${R}_{i,j}\sqrt{{\widehat{V}}_i{\widehat{V}}_j}$, where ${\widehat{V}}_i$ and ${\widehat{V}}_j$ are the estimated variances of ${\widehat{\beta}}_{GE_i}$ and ${\widehat{\beta}}_{GE_j}$, respectively. Because the correlation among association statistics can be well approximated by the correlation among genotypes [56], ${R}_{i,j}$ is the correlation coefficient of the genotypes at the *i*^th^ and *j*^th^ SNPs. ${\boldsymbol{V}}_{L\times L}$ incorporates the pairwise correlations among SNPs and therefore the pruning of SNPs in high LD is not necessary in ADABF (but is still recommended to reduce the computational burden).

Let the summary score from the *b*^th^ resampling be ${S}_k^{(b)}$, where $k=1,\cdots, L$ and $b=1,\cdots, B$. *P*-value is the probability of obtaining a statistic as extreme as or more extreme than the observed statistic under the null hypothesis. Therefore, the *P*-value of ${S}_k$ is estimated by $\frac{1}{B}{\sum}_{b=1}^BI\left({S}_k^{(b)}\ge {S}_k\right)$, where $k=1,\cdots, L$ and $I\left({S}^{(b)}_{k}\ge {S}_{k}\right)$ is an indicator variable with an outcome of 1 if ${S}_{k}^{(b)}\ge {S}_{k}$ or 0 if otherwise. Likewise, we calculate the *P*-value of ${S}_k^{(b)}$ by $\frac{1}{B-1}{\sum}_{b^\prime\ne b}I\left({S}_k^{\left({b}^{\prime}\right)}\ge {S}_k^{(b)}\right)$, where $k=1,\cdots, L$ and $b=1,\cdots, B$. Denote the minimum *P*-value (across $k=1,\cdots, L$) of the observed sample by $\min P$ and its counterparts from the *B* resampling replicates by $\min {P}^{(b)}$, $b=1,\cdots, B$. Therefore, the *P*-value of the ADABF test is $\frac{1}{B}{\sum}_{b=1}^BI\left(\min {P}^{(b)}\le \min P\right)$, which is compared with the usual nominal significance level of 0.05 (or 0.01). No multiple hypothesis correction is required because all the *L* SNPs are considered in an overall test. If the ADABF *P*-value is less than 0.05 (or 0.01), the null hypothesis of ${H}_0:{\beta}_{GE_1}=\!\!\cdots\!\! ={\beta}_{GE_L}=0$ is rejected, and we conclude that at least one SNP interacts with E. Because a *P*-value < 0.05 (or 0.01) leads to the rejection of *H_0_* of no polygenic SNP × E interactions [57], 1000 resampling replicates (*B* = 1000) is sufficient for our ADABF. This is different from the gene-based ADABF test where *P*-values are compared with the genome-wide significance level of $2.5\times {10}^{-6}= 0.05/20\,000$ (∼20 000 genes in the genome) [40]. Given a significant ADABF test, the subsequent step is to pinpoint which SNPs interact with E.

From the above resampling procedure, we can also obtain the resampling false discovery rate (FDR). In the *b*^th^ resampling replicate, we have ${BF}_1^{(b)},\cdots, {BF}_L^{(b)}$, for the *L* SNP × E. Based on the *B* resamples, the average number of false positives in a resampling replicate (while claiming significance of the leading *k* SNP × E) is estimated by ${FP}_{(k)} = \frac{1}{B}{\sum}_{b=1}^B{\sum}_{l=1}^LI\left({BF}_l^{(b)} \ge {BF}_{(k)}\right)$. The corresponding FDR is thus estimated as ${FDR}_{(k)}=\frac{1}{k}{FP}_{(k)}$, i.e. the number of false positives over the number of significant findings [58, 59]. We find the maximum *k* that satisfies ${FDR}_{(k)}<5\%$ and the SNPs corresponding to the leading *k* BFs are suggested to have interactions with E. The R code of the ADABF approach can be downloaded from http://homepage.ntu.edu.tw/∼linwy/ADABFGEPoly.html. We also provide the pipeline from PLINK to ADABF, at http://homepage.ntu.edu.tw/∼linwy/ADABFGEPolyPLINK.html.

The Gaussian (normal) prior is the most common choice for a prior distribution [52, 60]. Under this setting, Wakefield has derived a simple and convenient BF formula, as shown in Equation (3) [55]. It is hard to justify that the prior really follows a normal distribution. However, deviation from the Gaussian prior may not make much difference to ADABF results, because this method makes inference through the resampling procedure. For the observed data and for each of the resampling replicates, we obtain BFs according to the same prior. As shown by the following simulations, ADABF performs well although the true interaction effects (${\beta}_{GE_l}$s) never really come from a Gaussian distribution.

### GRS based on marginal effects of SNPs

We compare ADABF with GRS-M and GRS-I. Regarding GRS-M, the phenotype is first regressed on each of the *L* SNPs, as shown by Equation (1). The regression coefficients (${\widehat{\beta}}_{G_l}$s) of the SNPs that are more associated with the phenotype (*P*-value less than a certain threshold) are treated as the weights of the GRS. To be specific, the pre-scaled GRS-M of the *i*^th^ subject is defined as follows:

(4) \begin{equation*} {GRS}_{Mi,t}^{pre}={\sum}_{l =1}^L{\widehat{\beta}}_{G_l}{G}_{il}I\left({P}_{G_l} \lt{P}_t\right)\!,i = 1,\cdots, n;t = 1,\cdots, 10, \end{equation*}

where ${\widehat{\beta}}_{G_l}$ is estimated by the GLM in Equation (1), ${G}_{il}$ is the number of minor alleles at the *l*^th^ SNP of the *i*^th^ subject, $I\left(\cdot \right)$ is the indicator variable, ${P}_{G_l}$ is the *P*-value of testing ${H}_0:{\beta}_{G_l}=0$ versus ${H}_1:{\beta}_{G_l}\ne 0$ and ${P}_t$ is the *t*^th^*P*-value threshold. Most investigators use a *P*-value threshold to select a subset of SNPs for a GRS [20, 22, 57, 61–64]. We used 10 thresholds to explore the strength of GRS: 0.0001, 0.00025, 0.0005, 0.001, 0.0025, 0.005, 0.01, 0.025, 0.05 and 0.1.

For example, if the phenotype is binary (*Y* = 1 means diseased whereas *Y* = 0 indicates non-diseased) and the minor allele at the *l*^th^ SNP is a risk allele, ${\widehat{\beta}}_{G_l}$ estimated by the GLM (i.e. a logistic regression) in Equation (1) will be positive. Subjects with more minor alleles at this SNP will get an increase in their ${GRS}_{Mi,t}^{pre}$. However, if the minor allele at the *l*^th^ SNP is a protective allele, ${\widehat{\beta}}_{G_l}$ will be negative and subjects with more minor alleles at this SNP will get a decrease in their ${GRS}_{Mi, t}^{pre}$. Therefore, summing the information of the *L* SNPs, a larger ${GRS}_{Mi,t}^{pre}$ indicates a larger disease liability. ${GRS}_{Mi,t}^{pre}$ is then rescaled to calibrate the number of phenotype-increasing alleles [18]:

(5) \begin{equation*} {GRS}_{Mi, t}=\frac{GRS_{Mi, t}^{pre}\kern0.5em \times \kern0.5em number\;of\;available\;SNPs}{sum\;of\left|{\widehat{\beta}}_{G_l}\right| of\;available\;SNPs}. \end{equation*}

Denote $WA = {GRS}_{Mi, t}^{pre}\!\left/ \!\left( sum\;of\left|{\widehat{\beta}}_{G_l}\right| of\;available\;SNPs\right)\right.$ as the weighted average of the number of phenotype-increasing alleles. (For a set of values ${x}_i$s with nonnegative weights ${w}_i$s, the weighted average of this set is $\sum {w}_i{x}_i\!\left/ \!\sum {w}_i\right.$). The weights here, ${\widehat{\beta}}_{G_l}$s, can be positive or negative. Nonetheless, positive and negative ${\widehat{\beta}}_{G_l}$s will not be cancelled out in the formula of ${GRS}_{Mi,t}^{pre}$. As explained in the previous paragraph, regardless of the sign of each ${\widehat{\beta}}_{G_l}$, a larger ${GRS}_{Mi, t}^{pre}$ indicates a larger disease liability. Therefore, the denominator of *WA* is the sum of the absolute values of ${\widehat{\beta}}_{G_l}$s.

Because ${\widehat{\beta}}_{G_l}$s can be positive or negative, ${GRS}_{Mi, t}^{pre}$ and *WA* can be positive or negative as well. The range (maximum–minimum) of *WA* is up to 2, i.e. the number of minor alleles at each SNP. (For a set of values ${x}_i$s $\in \left\{0,1,2\right\}$ with nonnegative weights ${w}_i$s, the weighted average of this set is $\sum {w}_i{x}_i\!\left/ \!\sum {w}_i\right.$, ranging from 0 to 2.) To reflect the number of phenotype-increasing alleles from multiple loci, *WA* is multiplied by the number of available SNPs, as shown in Equation (5) [18].

Given the *t*^th^*P*-value threshold ($t=1,\cdots, 10$), we calculate ${GRS}_{Mi, t}$ for all the *n* subjects, fit the following GLM, and test ${H}_0:{\phi}_{GE}=0$ versus ${H}_1:{\phi}_{GE}\ne 0$:

(6) \begin{equation*} g\left[E\left({Y}_i\right)\right]={\phi}_0+{\phi}_G{GRS}_{Mi,t}+{\phi}_E{E}_i+{\phi}_{GE}{GRS}_{Mi,t}\cdot {E}_i+{\boldsymbol{\phi}}_X^{\prime }{X}_i,i=1,\cdots, n. \end{equation*}

Because we consider 10 *P*-value thresholds, 10 GLMs are fitted and ${H}_0:{\phi}_{GE}=0$ is tested 10 times.

In Equation (6), ${GRS}_{Mi,t}$ is in the same scale as the number of phenotype-increasing alleles [18], and therefore the regression coefficient ${\phi}_{GE}$ can be explained as follows. For continuous traits, each additional trait-increasing allele is associated with ${\phi}_{GE}$ change in trait for subjects with ${E}_i=1$ than for subjects with ${E}_i=0$. For binary traits, each additional disease susceptibility allele is associated with an OR of $\exp \left({\phi}_{GE}\right)$ for subjects with ${E}_i=1$ than for subjects with ${E}_i=0$.

### GRS based on SNP × E interactions

Hüls *et al*. have proposed the ‘GRS-interaction-training approach’ [28]. This is the 1st study presenting GRS with weights from the SNP × E interaction term itself. This approach was originally designed for pathway-oriented G × E studies. Borrowing the concept of ‘GRS-interaction-training approach’ [28], we construct a GRS according to the weights from the SNP × E interaction term itself. Different from the ‘GRS-interaction-training approach’ [28], we cannot fit a multivariate elastic net regression [36] on the typical large number of SNPs in GWAS. Instead, we estimate the SNP × E interaction effects by respective GLMs, as shown in the following Equation (7).

We first randomly split the whole sample into a training subset and a testing subset and an even split (1:1) is expected to yield the greatest power for GRS-I [22, 28]. Suppose the sample sizes of the training subset and the testing subset are ${n}_1$ and ${n}_2$, (${n}_1 \approx {n}_2$), respectively. We used the training subset to regress the phenotype on each SNP, E and SNP × E. The GLM for the *l*^th^ SNP ($l = 1,\cdots, L$) is as follows:

(7) \begin{equation*} g\left[E\left({Y}_i\right)\right]={\beta}_0+{\beta}_{G_l}{G}_{il}+{\beta}_E{E}_i+{\beta}_{GE_l}{G}_{il}{E}_i+{\boldsymbol{\beta}}_X^{\prime }{X}_i,i=1,\cdots, {n}_1. \end{equation*}

The pre-scaled GRS-I of the *i*^th^ subject in the testing subset is as follows:

(8) \begin{equation*} {GRS}_{Ii\, , t}^{pre} = {\sum}_{l=1}^L{\widehat{\beta}}_{GE_l}{G}_{il}I\left({P}_{GE_l} \lt {P}_t\right),\kern0.5em i = 1,\cdots, {n}_2, \end{equation*}

where ${\widehat{\beta}}_{GE_l}$ has been estimated by the GLM in Equation (7) and ${P}_{GE_l}$ is the *P*-value of testing ${H}_0:{\beta}_{GE_l} = 0$ versus ${H}_1:{\beta}_{GE_l} \ne 0$ using the training subset.

For example, if the minor allele at the *l*^th^ SNP has a synergistic effect with E on the phenotype, ${\widehat{\beta}}_{GE_l}$ estimated by the GLM in Equation (7) will be positive. Subjects with more minor alleles at this SNP will get an increase in their ${GRS}_{Ii, t}^{pre}$. In contrast, if the minor allele at the *l*^th^ SNP has an antagonistic effect with E on the phenotype, ${\widehat{\beta}}_{GE_l}$ will be negative and subjects with more minor alleles at this SNP will get a decrease in their ${GRS}_{Ii,t}^{pre}$. Therefore, summing the information of the *L* SNPs, a larger ${GRS}_{Ii,t}^{pre}$ indicates that the genetic makeup has a larger synergistic effect with E on the phenotype. ${GRS}_{Ii, t}^{pre}$ is then rescaled to calibrate the number of alleles exhibiting synergistic effects with E on the phenotype [18]:

(9) \begin{equation*} {GRS}_{Ii, t} =\frac{GRS_{Ii, t}^{pre}\kern0.5em \times \kern0.5em number\;of\;available\;SNPs}{sum\;of\left|{\widehat{\beta}}_{GE_l}\right| of\;available\;SNPs}. \end{equation*}

Given the *t*^th^*P*-value threshold ($t = 1,\cdots, 10$), we calculate ${GRS}_{Ii,\kern0.5em t}$ for the ${n}_2$ subjects in the testing subset, fit the following GLM and test ${H}_0: {\psi}_{GE} =0$ versus ${H}_1 : {\psi}_{GE} \ne 0$:

(10) \begin{equation*} g\left[E\left({Y}_i\right)\right]={\psi}_0+{\psi}_G{GRS}_{Ii,t}+{\psi}_E{E}_i+{\psi}_{GE}{GRS}_{Ii,t}\cdot {E}_i+{\boldsymbol{\psi}}_X^{\prime }{X}_i,i=1,\cdots, {n}_2. \end{equation*}

Because we consider 10 *P*-value thresholds, 10 GLMs are fitted and ${H}_0:{\psi}_{GE}=0$ is tested 10 times.

In Equation (10), ${GRS}_{Ii, t}$ is in the same scale as the number of alleles exhibiting synergistic effects with E [18] and therefore the regression coefficient ${\psi}_{GE}$ can be explained as follows. For continuous traits, each additional allele exhibiting synergistic effects with E is associated with ${\psi}_{GE}$ change in trait for subjects with ${E}_i = 1$ than for subjects with ${E}_i =0$. For binary traits, each additional allele exhibiting synergistic effects with E is associated with an OR of $\exp \left({\psi}_{GE}\right)$ for subjects with ${E}_i = 1$ than for subjects with ${E}_i=0$.

The data-splitting strategy is required to preserve the type I error rates of GRS-I [22, 28]. However, we do not need to split the whole sample into two subsets when performing GRS-M, because the SNPs are screened according to their marginal associations with the phenotype (rather than the strength of SNP × E). Corollary 1 proposed by Dai *et al.* [43] has justified the validity of using marginal associations as the screening test. Moreover, the following results of empirical type I error rates also verify that the data-splitting strategy is not required for GRS-M.

### Numerical experiments and simulations

TWB GWAS data were used for our simulations to consider real human LD patterns. The TWB aims at building a database that integrates the genomic data and lifestyles of residents aged 30–70 years in Taiwan [65]. Our study included 16555 unrelated community-based volunteers, among which 8213 were males and 8342 were females. This study was approved by the Research Ethics Committee of National Taiwan University Hospital (NTUH-REC no. 201612188RINA).

We removed SNPs with genotyping rates <95%, with Hardy–Weinberg test *P* < $5.7 \times {10}^{-7}$ [52], or with minor allele frequencies <1%. In total, 601 531 autosomal SNPs remained after removing SNPs that could not pass these quality control tests. These 601531 autosomal SNPs that passed the quality control stage were used to construct the principal components (PCs) to adjust for population stratification.

In order to eliminate a large degree of redundancy in SNPs and compare our ADABF with the GRS approaches, we removed SNPs in high LD [42] according to the pruning stage described in the ‘Methods’ section. After this pruning stage, 143 574 SNPs remained.

Then, we obtained a subset of SNPs that passed the screening stage by regressing diastolic blood pressure (DBP) on each of the 143574 SNPs while adjusting for sex, age, BMI and the 1st seven PCs (the reason of considering the 1st seven PCs can be seen in the section of ‘Application to TWB data’). There were 7652 SNPs with larger marginal effects on DBP (*P* < 0.05). The genotypes of the 16 555 subjects at these 7652 SNPs were used as our simulation materials. Moreover, without losing generality, we used smoking status as our E. Among the 16 555 subjects, 4104 subjects (∼24.8%) smoked for over 6 months, whereas 12 429 subjects did not. A total of 22 subjects did not respond to this question. We created a binary environmental exposure E, which was coded as 1 if the subject smoked for over 6 months and as 0 otherwise.

### Type I error rates

We assessed the type I error rates by assuming a disease with a prevalence of 5% and generating binary traits based on the following model:

(11) \begin{equation*} \log \frac{\Pr \left(Y=1\right)}{1-\Pr \left(Y=1\right)}=\log \frac{0.05}{1-0.05}=-2.94. \end{equation*}

The continuous traits were simulated by the following model:

(12) \begin{equation*} Y=e, \end{equation*}

where *e* was the random error term following the standard normal distribution.

### Power (in the absence of SNP main effects)

We evaluated the power performance of ADABF and GRS by randomly selecting *D* SNPs and letting them interact with E, where *D* = 20 or 50. The following three situations were considered: (1) 20 SNP × E with smaller effect sizes, (2) 20 SNP × E with larger effect sizes and (3) 50 SNP × E with smaller effect sizes.

The binary traits were simulated according to the following model:

(13) \begin{equation*} \log \frac{\Pr \left(Y=1\right)}{1-\Pr \left(Y=1\right)}=-2.94+\sum \limits_{d=1}^D{\beta}_{GE_d}{G}_dE. \end{equation*}

We let 50% of ${\beta}_{GE_d}$s be positive and 50% of ${\beta}_{GE_d}$s be negative. $\left|{\beta}_{GE_d}\right|$s (*d* = 1, …, *D*) were uniformly drawn from the intervals [log(1.2), log(1.4)] and [log(1.4), log(1.6)] for smaller effect sizes and larger effect sizes, respectively.

**Figure 1:**
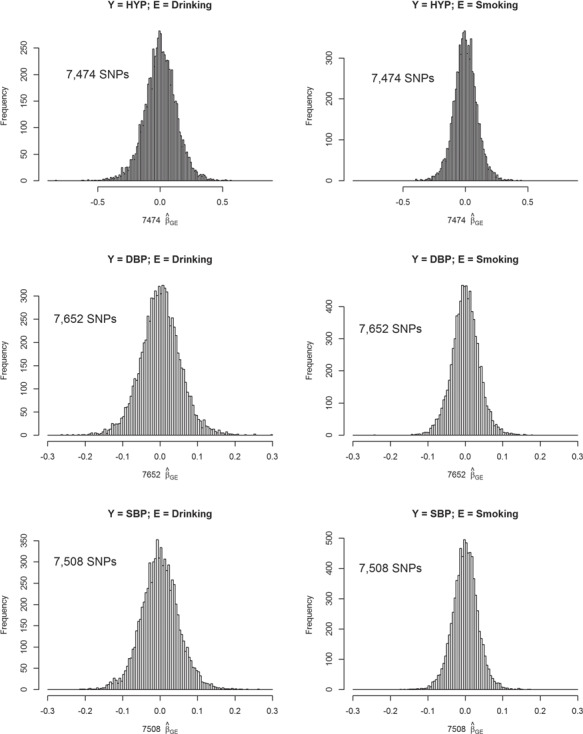
Histograms of ${\widehat{\beta}}_{GE}$s for SNPs that passed the pruning and screening stages. In total, 7474, 7652 and 7508 SNPs passed the pruning and screening stages for analyses of HYP (a binary trait), DBP and SBP, respectively. These SNPs were analyzed according to Equation (2), in which age, gender, BMI and the 1st seven PCs were adjusted. Two binary Es including alcohol drinking (left column) and smoking (right column) were considered. Here we show the histograms of ${\widehat{\beta}}_{GE}$s from the GLM in Equation (2).

Our setting of *D* (20 or 50) and the size of ${\beta}_{GE_d}$ [from log(1.2) to log(1.6)] in Equation (13) is reasonable for a GWAS. Take a binary trait—hypertension (HYP) in TWB as an example, which is defined as DBP > 80 mm Hg or systolic BP (SBP) > 130 mm Hg [66]. Totally 7474 SNPs that passed the pruning and screening stages were analyzed according to Equation (2), in which age, gender, BMI and the 1st seven PCs were adjusted. We considered two binary Es—alcohol drinking (described in the following ‘Application to TWB data’) and smoking. In total, 127 $\left|{\widehat{\beta}}_{GE}\right|$s > log(1.4) when E = drinking and 22 $\left|{\widehat{\beta}}_{GE}\right|$s > log(1.4) when E = smoking; 1064 $\left|{\widehat{\beta}}_{GE}\right|$s > log(1.2) when E = drinking and 405 $\left|{\widehat{\beta}}_{GE}\right|$s > log(1.2) when E = smoking. Approximately 50% of these ${\widehat{\beta}}_{GE}$ s were positive and 50% of ${\widehat{\beta}}_{GE}$s were negative. Histograms of ${\widehat{\beta}}_{GE}$s can be seen from the top row of Figure 1.

Continuous traits were generated by the following model:

(14) \begin{equation*} Y=\sum \limits_{d=1}^D{\beta}_{GE_d}{G}_dE+e, \end{equation*}

where *e* was the random error term following the standard normal distribution. We let 50% of ${\beta}_{GE_d}$s be positive and 50% of ${\beta}_{GE_d}$s be negative. $\left|{\beta}_{GE_d}\right|$s (*d* = 1, …, *D*, where *D* = 20 or 50) were uniformly drawn from the intervals [0.05, 0.07] and [0.07, 0.09] for smaller effect sizes and larger effect sizes, respectively. Likewise, the abovementioned situations (1–3) were accordingly considered in the simulations of continuous traits.

Our assumption of *D* and the size of ${\beta}_{GE_d}$ (from 0.05 to 0.09) in Equation (14) is also reasonable for continuous traits. Take DBP in TWB as an example. We first standardized DBP by $DB{P}^{\prime }=\left( DBP-\overline{DBP}\right)/ SD(DBP)$, where $\overline{DBP}$ and $SD(DBP)$ were the mean and the SD of DBP, respectively. Through this standardization, $DB{P}^{\prime }$ was in the similar scale with *Y* in Equation (14), where *e* was simulated from the standard normal distribution. In total, 7652 SNPs that passed the screening stage were analyzed according to Equation (2), in which age, gender, BMI and the 1st seven PCs were adjusted. A total of 1315 $\left|{\widehat{\beta}}_{GE}\right|$s > 0.07 when E = drinking and 451 $\left|{\widehat{\beta}}_{GE}\right|$s > 0.07 when E = smoking. Approximately 50% of these ${\widehat{\beta}}_{GE}$s were positive and 50% of ${\widehat{\beta}}_{GE}$s were negative. Histograms of ${\widehat{\beta}}_{GE}$s are presented in the middle row of Figure 1.

Similarly, we obtained the standardized $SB{P}^{\prime }$ and analyzed 7508 SNPs that passed the screening stage. In total, 1167 $\left|{\widehat{\beta}}_{GE}\right|$s > 0.07 when E = drinking and 353 $\left|{\widehat{\beta}}_{GE}\right|$s > 0.07 when E = smoking. Among these stronger ${\widehat{\beta}}_{GE}$s, ∼50% of them were negative. Histograms of ${\widehat{\beta}}_{GE}$s can be found from the bottom row of Figure 1. Therefore, in our simulation, the number of *D* (20 or 50) and the size of ${\beta}_{GE}$ are reasonable and modest.

### Power (in the presence of SNP main effects)

We then evaluated the power performance of the polygenic approaches in the presence of SNP main effects. The binary traits were simulated according to the following model:

(15) \begin{equation*} \log \kern0.5em \frac{\Pr \left(Y\ = 1\right)}{1-\Pr \left(Y = 1\right)}= -2.94\, + \sum \limits_{d=1}^D{\beta}_{G_d}{G}_d+ \sum \limits_{d=0.5D+1}^{1.5D}{\beta}_{GE_d}{G}_dE, \end{equation*}

$\left|{\beta}_{G_d}\right|$ *s* (*d* = 1, …, *D*) and $\left|{\beta}_{GE_d}\right|$*s* (*d* = 0.5*D* + 1, …, 1.5*D*, where *D* = 20 or 50) were uniformly drawn from the interval [log(1.2), log(1.4)] for smaller effect sizes, and were uniformly drawn from [log(1.4), log(1.6)] for larger effect sizes.

The continuous traits were simulated according to the following model:

(16) \begin{equation*} Y = \sum \limits_{d=1}^D{\beta}_{G_d}{G}_d + \sum \limits_{d= 0.5D+1}^{1.5D}{\beta}_{GE_d}{G}_dE+ e, \end{equation*}

where *e* was the random error term following the standard normal distribution. $\left|{\beta}_{G_d}\right|$s (*d* = 1, …, *D*) and $\left|{\beta}_{GE_d}\right|$s (*d* = 0.5*D* + 1, …, 1.5*D*, where *D* = 20 or 50) were uniformly drawn from [0.05, 0.07] for smaller effect sizes and were uniformly drawn from [0.07, 0.09] for larger effect sizes.

As expressed by Equations (15) and (16), we assume that SNPs 1 ∼ 0.5*D* present only main effects, SNPs (0.5*D* + 1) ∼ *D* exhibit both main effects and SNP × E interactions, and SNPs (*D* + 1) ∼ 1.5*D* exhibit only SNP × E interactions on traits. According to our observation from real data analyses (Figure 1), we let 50% of ${\beta}_{GE_d}$s be positive and 50% of ${\beta}_{GE_d}$s be negative. Moreover, we found minor alleles could be trait increasing or trait decreasing in real data analyses and therefore 50% of ${\beta}_{G_d}$s were assumed to be positive and 50% of ${\beta}_{G_d}$s were assumed to be negative. Among the SNPs that exhibit both main effects and SNP × E interactions, we let ${\beta}_{G_d}\cdot {\beta}_{GE_d}>0$ for ∼50% SNPs and ${\beta}_{G_d}\cdot {\beta}_{GE_d}<0$ for the remaining ∼50% SNPs, where *d* = 0.5*D* + 1, …, *D*.

## Results

### Type I error rates

In GWAS, a stringent genome-wide significance level ($5\times {10}^{-8}$) is typically used due to multiple hypothesis correction. The polygenic approaches investigated here combine SNPs across the genome in one test, and therefore, no multiple hypothesis correction is required. Table 1 presents the empirical type I error rates under a nominal significance level of 0.05 or 0.01, based on 10 000 replications of the binary traits and continuous traits separately. All the tests preserved the type I error rates. This simulation result confirms that the data-splitting strategy is not required for GRS-M or ADABF.

**Table 1:** Empirical type I error rates in the simulation study

**Traits**	**Nominal significance levels**	**ADABF**	***P*-value threshold (** ${P}_{t} $ **)**											
				**0.0001**	**0.00025**	**0.0005**	**0.001**	**0.0025**	**0.005**	**0.01**	**0.025**	**0.05**	**0.1**	M^*^ or I^*^
**Binary**	**0.05**	0.0466	GRS-M	0.0494	0.0510	0.0497	0.0524	0.0514	0.0539	0.0439	0.0485	0.0446	0.0468	0.0363
			GRS-I	0.0534	0.0560	0.0557	0.0491	0.0439	0.0446	0.0514	0.0488	0.0551	0.0507	0.0351
	**0.01**	0.0086	GRS-M	0.0135	0.0116	0.0087	0.0098	0.0088	0.0090	0.0076	0.0100	0.0083	0.0083	0.0059
			GRS-I	0.0085	0.0123	0.0115	0.0108	0.0107	0.0112	0.0088	0.0085	0.0134	0.0107	0.0076
**Continuous**	**0.05**	0.0508	GRS-M	0.0470	0.0480	0.0499	0.0434	0.0507	0.0495	0.0488	0.0522	0.0480	0.0484	0.0357
			GRS-I	0.0486	0.0509	0.0523	0.0570	0.0488	0.0501	0.0509	0.0490	0.0515	0.0476	0.0386
	**0.01**	0.0105	GRS-M	0.0077	0.0105	0.0079	0.0087	0.0093	0.0100	0.0101	0.0094	0.0111	0.0105	0.0077
			GRS-I	0.0087	0.0127	0.0110	0.0126	0.0108	0.0095	0.0114	0.0104	0.0101	0.0119	0.0075

Empirical type I error rates of ADABF, the GRS based on marginal associations (GRS-M), and the GRS based on interaction effects (GRS-I) are shown. Each GRS is evaluated at ten *P*-value thresholds: 0.0001, 0.00025, 0.0005, 0.001, 0.0025, 0.005, 0.01, 0.025, 0.05, and 0.1. Each entry represents the proportion of *P*-values smaller than the corresponding nominal significance level based on 10,000 simulation replications. M^*^and I^*^are GRS-M and GRS-I corrected for multiple testing, respectively. The *P*-value of M^*^(or I^*^) is ten times the minimum *P*-value of the ten GRS-M (or GRS-I) tests. An M^*^(or I^*^) test is claimed to be significant if its *P*-value < 0.05 or 0.01 (the nominal significance level).

For GRS, in addition to the type I error rates under 10 *P*-value thresholds, respectively, we also present its type I error rate while considering the 10 *P*-value thresholds simultaneously and then correcting for multiple testing. In Table 1, M^*^ and I^*^ are GRS-M and GRS-I corrected for multiple testing, respectively. According to the Bonferroni correction (BON), the *P*-value of M^*^ (or I^*^) is 10 times the minimum *P*-value of the 10 GRS-M (or GRS-I) tests. An M^*^ (or I^*^) test is claimed to be significant if its *P*-value < 0.05 or 0.01 (the nominal significance level). The type I error rates of M^*^ and I^*^ are smaller than the nominal significance levels because of the conservative nature of the BON.

### Power

Besides ADABF, GRS-M and GRS-I, we also evaluated the power performance of single marker analysis while controlling the family-wise error rate (FWER) at 5% using the BON, or controlling the FDR at 5% using the Benjamini–Hochberg approach (BH) [67]. Although BON and BH are not polygenic tests, they are also evaluated here because of their popularity. The power of BON or BH was calculated as the proportion of replications in which at least one SNP × E was identified.

**Figure 2:**
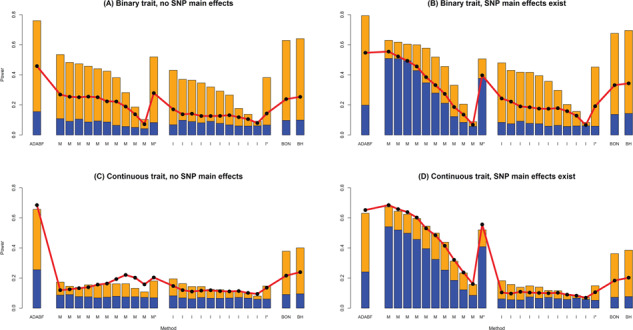
Empirical power given a nominal significance level of 0.05. The empirical power of ADABF, GRS-M (M) and GRS-I (I). GRS-M and GRS-I were evaluated at 10 *P*-value thresholds (from the left bar to the right bar): 0.0001, 0.00025, 0.0005, 0.001, 0.0025, 0.005, 0.01, 0.025, 0.05 and 0.1. M^*^ and I^*^ are GRS-M and GRS-I corrected for multiple testing, respectively. The *P*-value of M^*^ (or I^*^) is 10 times the minimum *P*-value of the 10 GRS-M (or GRS-I) tests. An M^*^ (or I^*^) test is claimed to be significant if its *P*-value < 0.05 (the nominal significance level). BON is single marker analysis while controlling the FWER at 5% using the BON; BH is single marker analysis while controlling the FDR at 5% using the BH. The height of the blue (yellow) bars marks the empirical power of each test given 20 SNP × E with smaller (larger) effect sizes. The red line with black points marks the empirical power given 50 SNP × E with smaller effect sizes.

Figure 2 presents the empirical power given the nominal significance level of 0.05, where the power of each scenario was calculated by 1000 replications. Our ADABF is more powerful than other methods in the absence of SNP main effects (Figure 2A and C). On the other hand, the presence of SNP main effects can considerably increase the power of GRS-M (Figure 2B and D). It constructs a GRS by aggregating the information of SNPs with stronger marginal effects. This approach becomes very powerful when some phenotype-associated SNPs also exhibit interactions with E.

**Figure 3:**
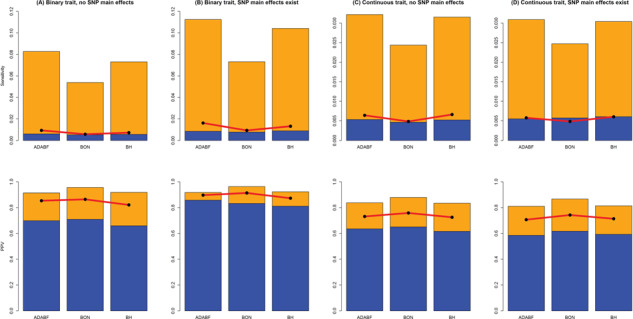
Sensitivity and PPV The sensitivity (the top row) is defined as the total number of true findings over the total number of SNP × E in the 1000 simulation replications, i.e. 20 000 or 50 000 (recall *D* = 20 or 50 in Equations 13–16). PPV (the bottom row) is defined as the total number of true findings over the total number of findings in the 1000 simulation replications. BON is single marker analysis while controlling the FWER at 5% using the BON; BH is single marker analysis while controlling the FDR at 5% using the BH. The height of the blue (yellow) bars marks the sensitivity/PPV of each method given 20 SNP × E with smaller (larger) effect sizes. The red line with black points marks the sensitivity/PPV given 50 SNP × E with smaller effect sizes.

Regarding the performance of pinpointing true SNP × E, we compared the sensitivity and PPV of our ADABF with BON and BH. Sensitivity is defined as the total number of true findings over the total number of SNP × E in the 1000 simulationreplications, i.e. 20000 or 50000 (recall *D* = 20 or 50 in Equations 13–16). PPV is defined as the total number of true findings over the total number of findings in the 1000 simulation replications. BON is the most conservative method, and therefore, it has the lowest sensitivity and the highest PPV (Figure 3). ADABF and BH performed similarly.

$PPV=1- FDP$, where FDP is the false discovery proportion. As shown in Figure 3, the FDPs are generally larger than the desired level of FDR, 5%. This is mostly because the FDR procedures assume that the statistics are unbiased [68]. Because fitting a multivariate regression on all SNPs is computationally infeasible, ADABF, BON and BH are all based onregression models that consider one SNP at a time (Equation 2). The statistics estimated from Equation 2 (${\widehat{\beta}}_{GE_l}$s) are biased because complex traits are usually influenced by multiple genetic variants, environmental exposures and the interplay between them (e.g. Equations 13–16). Therefore, the FDPs are larger than 5% for all the three methods (i.e. ADABF, BON and BH). The high FDP irrespective of the procedure used to correct for multiple testing has also been observed in regression-based analyses for environment-wide association study [68].

### Application to TWB data

We then applied these approaches to TWB data to explore SNP × alcohol and SNP × smoking interactions on BP. Approximately 85% of the TWB subjects were of the Han Chinese ancestry and ∼14.5% of the subjects belonged to a 3rd group that is genetically distinct from neighboring Southeast Asians [65].

In the TWB data, ‘drinking’ is defined as a weekly intake of greater than 150 cc of alcohol for at least 6 months. Among the 16 555 subjects, 1764 subjects (∼10.6%) answered ‘yes’ to alcohol drinking, whereas 14 779 subjects answered ‘no’. A total of 12 subjects did not respond to this question and were regarded as missing values. Smoking has been described in the section of ‘Numerical experiments and simulations’. Both alcohol drinking and smoking are binary Es. To obtain more reliable BP [69, 70], two measurements were taken with a 5 min rest interval and the average of the two measurements was recorded.

Regarding single marker analysis, for each of the 601 531 autosomal SNPs, we regressed DBP, SBP or HYP (yes versus no) on SNP, the E (i.e. alcohol drinking or smoking), SNP × E, while adjusting for age, gender, BMI and the 1st seven PCs. After adjusting for the 1st seven PCs, PLINK reported very low genomic inflation factors that were based on the median of the Chi-square statistics, i.e. ${\lambda}_{GC}= 1.00$ for both the DBP and SBP analyses and ${\lambda}_{GC}=1.02$ for HYP analysis. No significant SNP × E were found from the 601 531 autosomal SNPs, by controlling the FWER at 5% with the BON or by controlling the FDR at 5% with the BH [67].

**Figure 4:**
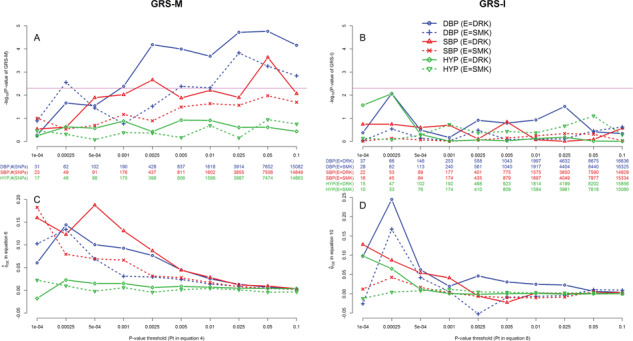
TWB analysis results using the GRS-M and GRS-I tests. The left and right columns show the GRS-M and GRS-I results, respectively. The black *x*-axes list the 10 *P*-value thresholds, i.e. ${P}_t$ in Equations (4) or (8). The blue (for DBP analysis), red (for SBP analysis) and green (for HYP analysis) *x*-axes list the number of SNPs used to construct ${GRS}_M$ or ${GRS}_I$. The *y*-axes of plots (A) and (B) are $-{\log}_{10}$(*P*-value of GRS-M) and $-{\log}_{10}$(*P*-value of GRS-I), respectively. Considering the 10 *P*-value thresholds used for the GRS, the Bonferroni-corrected significance level to control the FWER at 5% is $.05\!\left/ \!10\right.=.005$. The pink horizontal lines in plots (A) and (B) mark $-{\log}_{10}$(0.005) = 2.3. Moreover, ${\phi}_{GE}$ and ${\psi}_{GE}$ are estimated from Equations (6) and (10), respectively, and are shown in the *y*-axes of plots (C) and (D).

We then performed the GRS tests using the subset of SNPs that were nearly independent [22], i.e. the 143 574 SNPs that passed the pruning stage by the PLINK command shown in the section of ‘Methods’. Considering the 10 *P*-value thresholds used for the GRS, the Bonferroni-corrected significance level to control the FWER at 5% should be $0.05\!\left/ \!10\right.=0.005$. The pink horizontal line in Figure 4A marks the significance threshold, i.e. $-{\log}_{10}\,(0.005)\approx 2.3$. GRS-M identified SNP × alcohol interactions on DBP and SBP and SNP × smoking interactions on DBP (Figure 4A).

Figure 4C shows the regression coefficient ${\widehat{\phi}}_{GE}$ in Equation (6) at 10 *P*-value thresholds (${P}_t$). For example, when ${P}_t=5 \times {10}^{-4}$, ${GRS}_M$s of SBP, DBP and HYP analyses are computed by 91, 102 and 88 SNPs, respectively. Each additional SBP-increasing allele (DBP-increasing allele) is associated with ∼0.20 (∼0.10) mm Hg higher SBP (DBP) in drinkers than in nondrinkers. Each additional HYP susceptibility allele is associated with an OR of exp (0.015) = 1.015 in drinkers than in nondrinkers. Moreover, each additional SBP-increasing allele (DBP-increasing allele) is associated with ∼0.07 (∼0.07) mm Hg higher SBP (DBP) in smokers than in nonsmokers.

Finally, as described in the screening stage of the ‘Methods’ section, SNPs passing the screening at the desired significance level (*P* < .05) are then analyzed using ADABF. In the analysis of marginal associations, DBP, SBP and HYP were separately regressed on each of the 143 574 SNPs while adjusting for age, gender, BMI and the 1st seven PCs. Linear regression models were used for analyses of DBP and SBP, whereas logistic regression models were fitted for HYP. In total, 7652, 7508 and 7474 SNPs passed the screening test for DBP, SBP and HYP, respectively. Table 2 shows the analysis results based on 10^5^ resampling replicates in the ADABF approach. A *P*-value < 0.05 or 0.01 is sufficient to reject *H_0_* of no polygenic SNP × E interactions [57]. Like GRS-M (Figure 4A), ADABF identified SNP × alcohol interactions on DBP and SBP and SNP × smoking interactions on DBP, but ADABF provided more significant *P*-values than GRS-M. Additionally, ADABF identified SNP × alcohol interactions on HYP.

**Table 2:** TWB analysis results using the ADABF, BON, and BH approaches

	**ADABF** ^**1**^			**BON** ^**2**^		**BH** ^**3**^
***SNPxalcohol on DBP* (**based on 7,652 SNPs**)**						
***P-value***	$<0.00001$			---		---
**SNP found to have interaction with alcohol consumption**	rs10811568 (Resampling FDR = 1.2%)			rs10811568		rs10811568
***SNPxalcohol on SBP* (**based on 7,508 SNPs**)**						
***P-value***	$<0.00001$			---		---
**SNP found to have interaction with alcohol consumption**	rs62065089 (Resampling FDR = 0.4%)			rs62065089		rs62065089
***SNPxalcohol on HYP* (**based on 7,474 SNPs**)**						
***P-value***	$0.00098$			---		---
**SNP found to have interaction with alcohol consumption**	---			---		---
***SNPxsmoking on DBP* (**based on 7,652 SNPs**)**						
***P-value***	$0.00059$			---		---
**SNP found to have interaction with smoking**	rs79990035 (Resampling FDR = 1.1%)			rs79990035		rs79990035
***SNPxsmoking on SBP* (**based on 7,508 SNPs**)**					
***P-value***	0.1573			---		---
**SNP found to have interaction with smoking**	---			---		---
***SNPxsmoking on HYP* (**based on 7,474 SNPs**)**						
***P-value***	0.0592			---		---
**SNP found to have interaction with smoking**	---			---		---

^1^The *P*-value of ADABF and the resampling FDR were based on 10^5^ resampling replicates. In SNPxalcohol interaction analysis on DBP or SBP, the observed interaction signal was more significant than that of all the 10^5^ resampling replicates. Therefore, the *P*-values were represented as “$<0.00001$”. A *P*-value < 0.05 or 0.01 is sufficient to reject *H_0_* of no polygenic SNP$\times$E interactions [57]. No more resampling replicates are required to obtain a more precise *P*-value. *P*-values < 0.05 are highlighted.

^2^BON is single marker analysis while controlling the FWER at 5% using the Bonferroni correction.

^3^BH is single marker analysis while controlling the FDR at 5% using the Benjamini-Hochberg approach.

GRS-I did not provide any significant results under the Bonferroni-corrected significance level of $0.05\left/ \!10\right.\!=0.005$ (Figure 4B). The pink horizontal line in Figure 4B marks the significance threshold, i.e. $-{\log}_{10}\,(0.005)\approx 2.3$. This result was consistent with the above finding in our simulations. That is, GRS-I is the least powerful approach due to its data-splitting strategy.

Figure 4D shows the regression coefficient ${\widehat{\psi}}_{GE}$ in Equation (10) at 10 *P*-value thresholds (${P}_t$). Although GRS-I tests are not significant at all the 10 ${P}_t$s, we still explain the meaning of ${\widehat{\psi}}_{GE}$ here. For example, when ${P}_t=5\times {10}^{-4}$, ${GRS}_I$s of SBP (E = drinking and smoking) and DBP (E = drinking and smoking) analyses are computed by 89, 84, 146 and 113 SNPs, respectively. Each additional allele exhibiting synergistic effects with drinking is associated with ∼0.05 (∼0.05) mm Hg higher SBP (DBP) in drinkers than in nondrinkers. Moreover, each additional allele exhibiting synergistic effects with smoking is associated with ∼0.02 (∼0.04) mm Hg higher SBP (DBP) in smokers than in nonsmokers.

**Figure 5:**
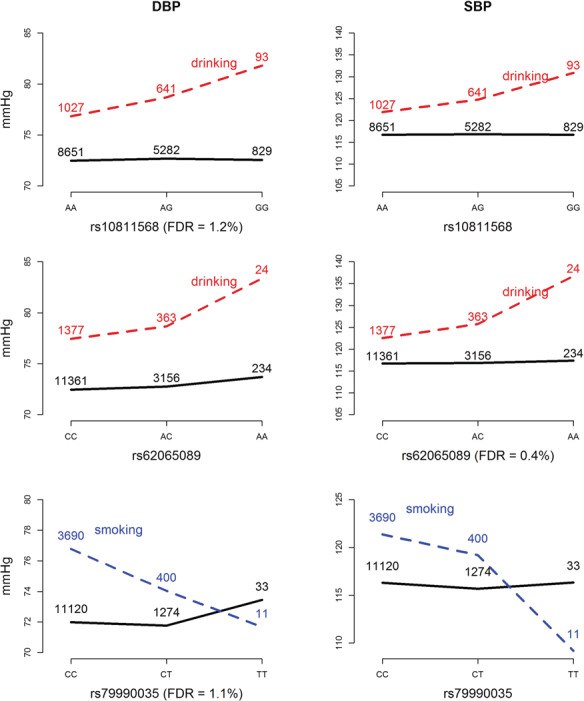
Plots of SNP × alcohol or SNP × smoking interaction effects on DBP and SBP Controlling the resampling FDR at 5%, we found an rs10811568 × alcohol interaction on DBP, rs62065089 × alcohol interaction on SBP and rs79990035 × smoking interaction on DBP. As shown in these plots, these identified interaction patterns in DBP/SBP are similar to those in SBP/DBP. The black curves depict the mean of DBP/SBP among the nondrinkers/nonsmokers, whereas the red/blue dashed curves depict the mean among the drinkers/smokers. The number shown on each point represents the sample size of that category.

We also identified an rs10811568 × alcohol interaction on DBP (resampling FDR = 1.2%), rs62065089 × alcohol interaction on SBP (resampling FDR = .4%) and rs79990035 × smoking interaction on DBP (resampling FDR = 1.1%) through the resampling procedure in ADABF. Table 3 lists the detailed information on these three SNPs. Figure 5 presents plots of these interaction effects.

**Table 3:** The three SNPs that interact with alcohol consumption or smoking

SNP	Chromosome	Position (base pair)	Mapped gene	Minor allele	Major allele	MAF	E	Phenotype	SNP × E interaction test^1^				
									${\widehat{\beta}}_{GE} $	$s.e.\left({\widehat{\beta}}_{GE}\right) $	Wald statistic	*P*-value	Bayes factor
rs10811568	9	21543444	*MIR31HG*	G	A	0.235	Alcohol	**DBP** ^**2**^	**1.9753**	**0.4125**	**4.788**	$1.7\times {10}^{-6} $	**11,875**
								SBP^3^	3.1164	0.6240	4.994	$6.0\times {10}^{-7} $	31,233
rs62065089	17	63843058	*CEP112*	A	C	0.122	Alcohol	DBP	1.4872	0.5403	2.753	0.00592	8
								**SBP** ^**4**^	**4.0673**	**0.8169**	**4.979**	$6.5\times {10}^{-7} $	**28,933**
rs79990035	2	54418123	*ACYP2*	T	C	0.053	Smoking	**DBP** ^**5**^	**-2.6929**	**0.5601**	**-4.808**	$1.5\times {10}^{-6} $	**12,827**
								SBP	-2.4950	0.8469	-2.946	0.00322	14

^1^DBP or SBP was regressed on each of the SNPs, the environmental factor (i.e., alcohol consumption or smoking), and SNP×E, while adjusting for age, gender, BMI, and the first seven principal components.

^2^The rs10811568×alcohol interaction was found in DBP analysis (the highlighted row).

^3^Because the marginal association of SNP rs10811568 with SBP was not significant (*P* > 0.05), this SNP was not in the screened subset of 7,508 SNPs. Therefore, it was not pinpointed from the SNP×alcohol interaction analysis on SBP.

^4^The rs62065089×alcohol interaction was found in SBP analysis (the highlighted row).

^5^The rs79990035×smoking interaction was found in DBP analysis (the highlighted row).

These three SNPs can also be identified by BON/BH when controlling the FWER/FDR at 5% given the ∼7600 SNPs that passed the pruning and screening stages. Because we have removed SNPs in high LD, these ∼7600 SNPs are nearly independent of each other and therefore the resampling FDR and BH lead to the same results. Despite this, performing ADABF is still worthwhile. As shown by Table 2, SNP × alcohol interactions on HYP can only be detected by the ADABF polygenic test, although no individual SNP × alcohol interactions can be identified from the subsequent resampling FDR. Nothing can be found if we bypass the ADABF polygenic test and directly use BON/BH. This result is consistent with the power gain of ADABF compared with BON/BH (Figure 2).

For a polygenic test, a *P*-value <0.05 or 0.01 is sufficient to reject *H_0_* of no polygenic effects [57]. Therefore, resampling 10^5^ replicates is sufficient for a real data analysis. ADABF takes ∼2.5 h to analyze SNP × E on a continuous phenotype based on a Linux platform with a Dell Intel Xeon E5–2690 2.9 GHz processor and 8 GB of memory. Approximately 3.4 h are required for the analysis of a binary phenotype, because fitting a logistic regression takes more computation time than fitting a linear regression.

## Discussion

Genetic effects can differ between subjects depending on their lifestyle factors or environmental exposure because of G × E [5]. Therefore, the identification of G × E is important for investigating new mechanisms in disease [71]. Given a specific sample size, the power to detect G × E is much lower than the power to detect genetic main effects [47, 72]. The exploration of G × E from GWAS data is even more challenging due to the harsh multiple-testing penalty. There is a great need to discover a powerful polygenic approach that can identify G × E and further pinpoint SNPs that interact with E.

Most complex diseases are polygenic (influenced by many small genetic effects), including obesity [73, 74], HYP [75], schizophrenia and bipolar disorder [76]. The GRS that aggregates multiple genetic variants into a score is widely used for testing and prediction [77, 78]. A majority of the G × E findings were discovered by the GRS approach [3, 5, 79–83]. For example, recently, Rask-Andersen *et al.* [5] constructed a GRS composed of 94 independent BMI-associated SNPs that were reported by a previous GWAS [32], and they found interactions between this GRS and several Es. However, an appropriate external GWAS may not be available for other phenotypes or other ethnicity.

When external information is unavailable, the weights for a GRS have to be determined internally. Because the ‘best’ *P*-value threshold for the ‘optimal’ subset of SNPs is unknown, many studies constructed a panel of GRSs under various *P*-value thresholds [21, 78, 84, 85]. Therefore, the significance of a GRS test has to be corrected by the number of *P*-value thresholds evaluated [57]. (Specifying a *P*-value threshold to select SNPs is not an issue in the ‘GRS-marginal-internal approach’ and ‘GRS-interaction-training approach’. Hüls *et al*. select SNPs according to a multivariate elastic net regression [28, 35].)

We here compare our ADABF with the GRS-M and GRS-I tests. Regarding the power to detect polygenic–environment interactions, ADABF is the most powerful test in the absence of SNP main effects (Figure 2A and C). When SNP main effects exist, GRS-M can outperform ADABF and can become the best test (Figure 2B and D). GRS-I is the least powerful approach due to its data-splitting strategy.

In most applications of BFs [52], specifying a different prior variance (*W*) will change the magnitude of a BF and can lead to a different inference. For example, a BF between 10 and 100 is regarded as a ‘strong’ evidence against *H_0_*, whereas a BF larger than 100 is deemed as a ‘decisive’ evidence against *H_0_* [86]. However, ADABF does not fully rely on the absolute magnitudes of BFs. Instead, ADABF compares the BFs from the observed data with those from the resampling replicates, under the same prior. Therefore, this method is robust to the setting of the prior variance (*W*) [40]. When *W* was set at .1^2^ = 0.01 or 0.3^2^ = 0.09, the ADABF results were very close to those obtained from *W* = 0.2^2^ = 0.04 (Supplementary Table S1 and 2).

When multiple SNPs interact with E but their effect sizes are small, we may obtain a significant ADABF test result; however, no individual SNP × E can be identified by controlling the resampling FDR at 5%. SNP × alcohol on HYP shown in Table 2 is an example. In this situation, if we bypass ADABF and directly use BH to identify SNP × E, we will find nothing, and G × E can be missed (comparing the power of ADABF and BH in Figure 2).

Gene × alcohol and gene × smoking interactions on BP have been found in Caucasians [49, 87] and Japanese [88, 89]. Our results support these G × E on BP in Han Chinese as well. Although ADABF is a powerful polygenic test for detecting G × E, the identification of individual SNP × E is still very challenging. If the ADABF test is significant (*P*-value < 0.05 or 0.01, no multiple hypothesis correction is required), we conclude that G × E interactions exist and the E can modify the genetic effect on the phenotype. However, each SNP × E test may not be able to reach a sufficient power that can withstand the multiple hypothesis correction. In this situation, GRS-M can help to identify whether synergistic or antagonistic interactions exist between risk alleles and E, according to the sign of ${\widehat{\phi}}_{GE}$ in Equation (6). For example, the positive ${\widehat{\phi}}_{GE}$s (Figure 4C) indicate that BP-increasing alleles elevate more BP in drinkers (smokers) than in nondrinkers (nonsmokers).

We found the rs79990035 × smoking interaction on DBP (resampling FDR = 1.1%). The SNP rs79990035 is located in the *acylphosphatase 2* (*ACYP2*) gene. Cheng *et al.* recently also found an *ACYP2* × smoking interaction related to susceptibility to ischemic stroke (IS) in a Han Chinese population [90]. High BP levels are usually observed in acute IS patients [91–93]. Therefore, for Han Chinese, the *ACYP2* × smoking interaction on BP warrants further investigation.

Some G × E have been discovered by constructing a GRS based on external GWAS results [3–5, 17–19, 29–31]. However, appropriate external information is not always available. This work compares polygenic approaches to identify G × E in the context of GWAS, when external information is unavailable. Using ADABF or GRS-M, many hidden G × E might be explored. Moreover, GRS-M can help to identify whether synergistic or antagonistic interactions exist between risk alleles and E.

**Key Points**

- The scientific community usually constructs a GRS and tests the interaction between this score and an E. However, until now, little has been known about the performance of the GRS for detecting G × E. Moreover, appropriate external weights required for a GRS are not always available.
- We explored a powerful polygenic approach for detecting G × E when external weights are not available, by comparing our ‘ADABF’ with the GRS-M and GRS-I effects.
- ADABF is the most powerful method in the absence of SNP main effects, whereas GRS-M is generally the best test when SNP main effects exist. GRS-I is the least powerful test due to its data-splitting strategy. This work provides guidance to choose a polygenic approach to detect G × E when external information is unavailable.

## Supplementary Material

bby086_Supp

## Notes

**Wan-Yu Lin** is an associate professor at the Institute of Epidemiology and Preventive Medicine & Department of Public Health, College of Public Health, National Taiwan University.

## Notes

**Ching-Chieh Huang** is a Masters student at the Institute of Epidemiology and Preventive Medicine, College of Public Health, National Taiwan University. He is under Wan-Yu Lin’s supervision.

## Notes

**Yu-Li Liu** is an investigator at the Center for Neuropsychiatric Research, National Health Research Institutes.

## Notes

**Shih-Jen Tsai** is the Chairman at the Department of Psychiatry, Taipei Veterans General Hospital and a professor at the Division of Psychiatry, National Yang-Ming University.

## Notes

**Po-Hsiu Kuo** is a professor at the Institute of Epidemiology and Preventive Medicine & Department of Public Health, College of Public Health, National Taiwan University.
