# Supplementary material for: Polygenic approaches to detect gene–environment interactions when external information is unavailable
Source: Brief Bioinform. 2018 Sep 13;20(6):2236–52. doi: 10.1093/bib/bby086 (PMC6954453; doi:10.1093/bib/bby086)
Supplement: bby086_Supp [file bby086_supp.pdf]

	(B) Binary trait, SNP main effects exist			(D) Continuous trait, SNP main effects exist		
	**ADABF** $W = 0.2^2 = 0.04$	**ADABF** $W = 0.1^2 = 0.01$	**ADABF** $W = 0.3^2 = 0.09$	**ADABF** $W = 0.2^2 = 0.04$	**ADABF** $W = 0.1^2 = 0.01$	**ADABF** $W = 0.3^2 = 0.09$
**Power**						
(1) 20 SNP × E with smaller effect sizes	0.198	0.201	0.190	0.240	0.250	0.232
(2) 20 SNP × E with larger effect sizes	0.793	0.785	0.791	0.630	0.642	0.622
(3) 50 SNP × E with smaller effect sizes	0.546	0.565	0.537	0.652	0.679	0.652
**Sensitivity**						
(1) 20 SNP × E with smaller effect sizes	0.008	0.008	0.008	0.006	0.006	0.006
(2) 20 SNP × E with larger effect sizes	0.113	0.107	0.112	0.031	0.032	0.031
(3) 50 SNP × E with smaller effect sizes	0.016	0.017	0.016	0.006	0.006	0.006
**PPV**						
(1) 20 SNP × E with smaller effect sizes	0.858	0.869	0.865	0.585	0.603	0.582
(2) 20 SNP × E with larger effect sizes	0.917	0.926	0.919	0.811	0.803	0.804
(3) 50 SNP × E with smaller effect sizes	0.897	0.895	0.912	0.707	0.718	0.702

Table S1. Power, sensitivity, and PPV of the ADABF approach with three levels of prior standard deviation (0.2, 0.1, 0.3)

	ADABF $W = 0.2^2 = 0.04$	ADABF $W = 0.1^2 = 0.01$	ADABF $W = 0.3^2 = 0.09$
SNPxalcohol on DBP **(based on 7,652 SNPs)**			
P-value	$< 10^{-5}$	$< 10^{-5}$	$< 10^{-5}$
**SNP found to have interaction with alcohol consumption**	rs10811568 (Resampling FDR = 1.2%)	rs10811568 (Resampling FDR = 1.0%)	rs10811568 (Resampling FDR = 1.3%)
SNPxalcohol on SBP **(based on 7,508 SNPs)**			
P-value	$< 10^{-5}$	$< 10^{-5}$	$< 10^{-5}$
**SNP found to have interaction with alcohol consumption**	rs62065089 (Resampling FDR = 0.4%)	rs62065089 (Resampling FDR = 0.5%)	rs62065089 (Resampling FDR = 0.4%)
SNPxalcohol on HYP **(based on 7,474 SNPs)**			
P-value	9.8×10^{-4}	1.1×10^{-3}	7.5×10^{-4}
**SNP found to have interaction with alcohol consumption**	---	---	---
SNPxsmoking on DBP **(based on 7,652 SNPs)**			
P-value	5.9×10^{-4}	8.2×10^{-4}	5.1×10^{-4}
**SNP found to have interaction with smoking**	rs79990035 (Resampling FDR = 1.1%)	rs79990035 (Resampling FDR = 1.2%)	rs79990035 (Resampling FDR = 0.9%)
SNPxsmoking on SBP **(based on 7,508 SNPs)**			
P-value	0.1573	0.1692	0.1541
**SNP found to have interaction with smoking**	---	---	---
SNPxsmoking on HYP **(based on 7,474 SNPs)**			
P-value	0.0592	0.0615	0.0568
**SNP found to have interaction with smoking**	---	---	---

Table S2. TWB analysis results using the ADABF approach with three levels of prior standard deviation (0.2, 0.1, 0.3)
The *P*-value of ADABF and the resampling FDR were based on 10^5 resampling replicates. *P*-values < 0.05 are highlighted.
